# Morphometry and skeletopy of kidneys and renal vessels in *Alouatta guariba clamitans* (Primates: Atelidae): case reports

**DOI:** 10.29374/2527-2179.bjvm005222

**Published:** 2023-05-18

**Authors:** Shirley Viana Peçanha, Thaís Mattos Estruc, Raquel Batista Junger de Carvalho, Carlos Augusto dos Santos-Sousa, Paulo Souza-Júnior, Marcelo Abidu-Figueiredo

**Affiliations:** 1 Veterinarian, BVM. Autonomous, Rio de Janeiro, RJ, Brazil.; 2 Biologist, MSc. Rio de Janeiro, RJ, Brazil.; 3 Veterinarian, MSc. Parque Nacional da Serra dos Órgãos, Teresópolis, Rio de Janeiro, RJ, Brasil, Brazil.; 4 Veterinarian, DSc. Centro de Ciências Biológicas e da Natureza, Universidade Federal do Acre (UFAC), Rio Branco, AC, Brazil.; 5 Veterinarian, DSc. Departamento de Medicina Veterinária, Universidade Federal do Pampa (UNIPAMPA), Uruguaiana, RS, Brazil.; 6 Veterinarian, DSc. Departamento de Anatomia Animal e Humana, Universidade Federal Rural do Rio de Janeiro (UFRRJ), Seropédica, RJ, Brazil.

**Keywords:** anatomy, red howler monkey, urinary system, vascularization, anatomia, bugio ruivo, sistema urinário, vascularização

## Abstract

Various animal models are used for research; however, non-human primates are well suited for biomedical research owing to their genetic homology with humans. The objective of this research was the anatomical characterization of red howler's kidneys in view of the scarcity of information in the literature. Protocols were approved by the Committee for Ethics in the Use of Animals at the Federal Rural University of Rio de Janeiro (number 018/2017). The study was conducted at the Laboratory of Teaching and Research in Domestic and Wild Animal Morphology, Federal Rural University of Rio de Janeiro. Specimens of *Alouatta guariba clamitans* were collected from the Serra dos Órgãos National Park road in Rio de Janeiro and subsequently frozen. Four adult cadavers (two males and two females) were used, identified, and injected with a 10% formaldehyde solution. Later, the specimens were dissected, and measurements and topography of the kidneys and renal vessels were recorded. The kidneys of *A. g. clamitans* resemble a “bean seed,” with a smooth surface. The longitudinal section shows two distinct regions, cortical and medullary; in addition, the kidneys are unipyramidal. The renal arteries emerged from the abdominal aorta as a single vessel posterior to the renal veins. The renal veins drained directly into the caudal vena cava as a single vessel in all specimens.

## Resumo

Vários modelos animais são usados para pesquisa, no entanto, primatas não humanos são adequados para pesquisas biomédicas devido à sua homologia genética com humanos. O objetivo desta pesquisa foi a caracterização anatômica dos rins do Bugio, tendo em vista a escassez de informações na literatura. Os protocolos foram aprovados pelo Comitê de Ética no Uso de Animais da Universidade Federal Rural do Rio de Janeiro (número 018/ 2017). O estudo foi realizado no Laboratório de Ensino e Pesquisa em Morfologia de Animais Domésticos e Silvestres da Universidade Federal Rural do Rio de Janeiro. Espécimes de *Alouatta guariba clamitans* foram coletados da estrada para o Parque Nacional da Serra dos Órgãos no Rio de Janeiro, e posteriormente congelados. Foram utilizados quatro cadáveres adultos (dois machos e duas fêmeas), identificados e injetados com solução de formaldeído a 10%. Os rins de *A. g. clamitans* assemelham-se a uma “semente de feijão”, com superfície lisa. Ao corte longitudinal apresenta duas regiões distintas, cortical e medular, além disso, apresenta-se unipiramidal. As artérias renais emergiram da aorta abdominal como um único vaso, posteriormente às veias renais. As veias renais drenaram diretamente para a veia cava caudal como um único vaso em todos os espécimes.

## Introduction

Using animals in biomedical research is recommended to improve and validate existing procedures, develop new products, and better understand different physiological and pathological processes. This is because no *in vitro* model can completely reproduce the complexity of the human organism. Animal models allow for the control of numerous variables that normally cannot be obtained in human studies (Fagundes & Taha, 2004).

Various animal models are used for research; however, non-human primates are well suited for biomedical research owing to their genetic homology with humans (Andrade et al., 2010; Auricchio, 2011). In biomedical research, interest in non-human primates has increased over that in other animals used in research laboratories (Ward & Vallender, 2012).

The Southern red howler monkey, *Alouatta guariba clamitans*, is found in the eastern part of Brazil, along the Atlantic Forest, in the states of Espírito Santo, Rio de Janeiro, Minas Gerais, São Paulo, Paraná, Santa Catarina, and Rio Grande do Sul, and also in the forest of Misiones Province in Argentina (Gregorin, 2006).

It belongs to the Atelidae family, is one of the largest neotropical primates, and feeds on a large number of leaves (Drubbel & Gautier, 1993). With an arboreal habitat, nearly every stratum of the forest has a tolerance for modifications/disturbances in the environment (Bicca-Marques, 2013; Silva & Bicca-Marques, 2013). Adult males of *A. g clamitans* weigh an average of 6.7 kg, with females weighing an average of 4.4 kg (Smith & Jungers, 1997).

The high density of human populations in the southern and southeastern regions of Brazil and the consequential destruction of their habitats have decreased the broad original distribution of *A. g. clamitans* to a few populations, restricted to isolated forestry fragments (Chiarello & Galetti, 1994; Crockett, 1998).

The main threats comprise the expansion of farming activities and urbanization, yellow fever epidemics, road kills on highways, accidents with power grids, and hunting (Almeida et al., 2012; Lokschin et al., 2007; Printes et al., 2001). For these reasons, the red howler monkey is considered a vulnerable species in most Brazilian states (Buss et al., 2021).

The objective of this research was the anatomical characterization of red howler's kidneys in view of the scarcity of information in the literature.

## Materials and methods

The protocols were approved by the Committee for Ethics in the Use of Animals in the Federal Rural University of Rio de Janeiro (number 018/2017).

The study was carried out in the Laboratory of Teaching and Research in Domestic and Wild Animal Morphology, Federal Rural University of Rio de Janeiro. Specimens of *A. g. clamitans* were collected from the Serra dos Órgãos National Park road in Rio de Janeiro, Brazil, and subsequently frozen. Samples with advanced signs of autolysis were excluded from the analysis. In this study, four adult cadavers of *A. g. clamitans*, two males and two females, with no evidence of renal alterations on macroscopic examination, were used. Initially, the animals were thawed in running water, sexed, and identified using a numbered plastic label. With the aid of a precision metal measuring tape, the body length of each animal was measured in centimeters, taking the vertex of the skull to the insertion of the tail as a reference.

The specimens were placed in the supine position, and the thoracic cavity was opened. The thoracic aorta was identified, dissected, and cannulated. The arterial system was irrigated with 0.9% sodium chloride and fixed intravascularly with 10% formaldehyde solution. After formaldehyde injection, latex (Petrolátex S-65, Refinery Duque de Caxias-REDUC-Petrobras, Duque de Caxias-RJ) was diluted 1:1 with red dye (Xadrez®) and injected through the thoracic aorta. Finally, the cadavers were submerged in 10% formaldehyde to complete fixation. After at least 7 days of fixation, the peritoneal cavity of the cadavers was opened, and the kidneys and renal vessels were identified and freed. Next, the location of the kidney in the vertebra was determined. Part of the hypaxial musculature was removed until the articulation of the last pair of ribs with the last thoracic vertebra and lumbar transverse processes was identified. The locations of the cranial and caudal poles of each kidney were identified and matched with the vertebral anatomy.

A caliper (0–150 mm, 0.01-mm resolution, accuracy ± 0.02 mm, Eda®) was used to measure the dimensions of the kidneys (length, width, thickness, and volume of the ellipsoid) and the length of their vessels in centimeters. Renal length was measured between the cranial and caudal ends of the kidney. The renal width was measured between the renal hilum and the midpoint of the lateral margin of the kidney. To measure renal thickness, the midpoint between the ventral face and the midpoint of the dorsal face of the kidney was used as a reference. The ellipsoid volume of each kidney was calculated using the equation length × width × thickness × 0.5236 as previously established (Sampaio, 1995). Vessel length was obtained for the renal artery and vein, with the measurement based on the renal hilum and abdominal aorta.

Data were tabulated in spreadsheets and expressed as arithmetic means and standard deviations. The nomenclature was based on the International Committee on Veterinary Gross Anatomical Nomenclature (2017).

## Case reports

**Figure 1:**
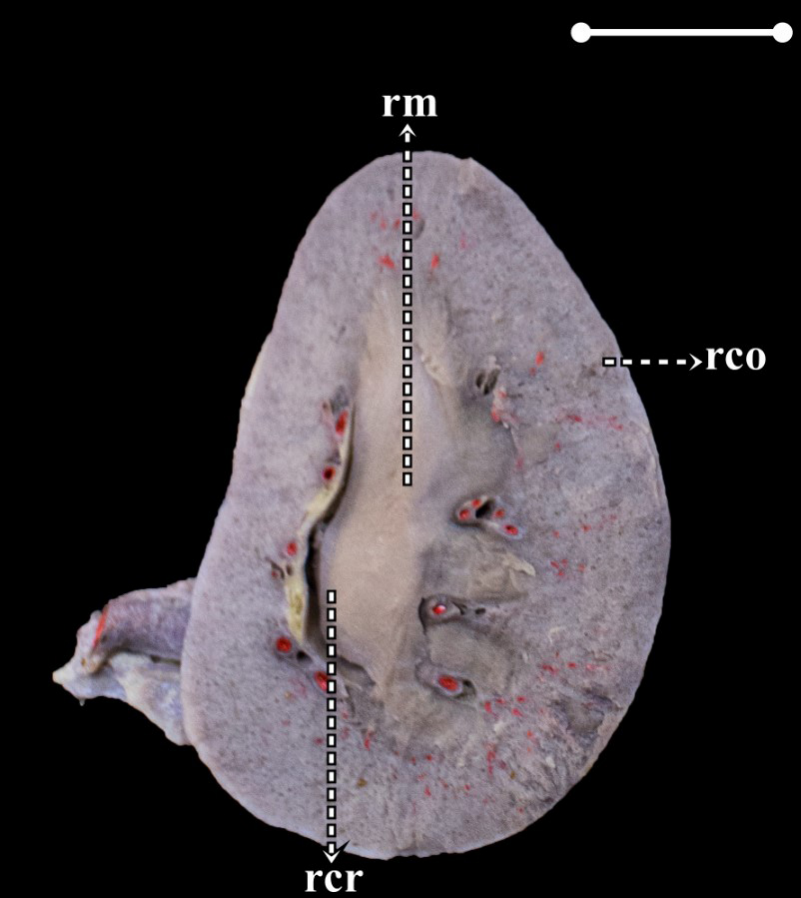
Digital photomacrography in a longitudinal section of the kidney in a male specimen of *Alouatta guariba clamitans*. rm, renal medulla; rco, renal cortex; rcr, renal crest. Scale bar: 1.0 cm.

In the specimens studied, the average body length was 46.00 ± 1.4 cm in males and 32.0 ± 1.4 cm in females. The kidneys of the *A. g. clamitans* were symmetrical in shape, resembled a “bean seed,” and also pale brown with a smooth surface. In the longitudinal section, the kidney consisted of a paler outer region, renal cortex, darker inner region, and medulla. The medullary region is simple and consists of a single pyramid, one renal crest, and renal pelvis. The ureters originated from the lower portion of the renal pelvis (Figure 1).

**Figure 2:**
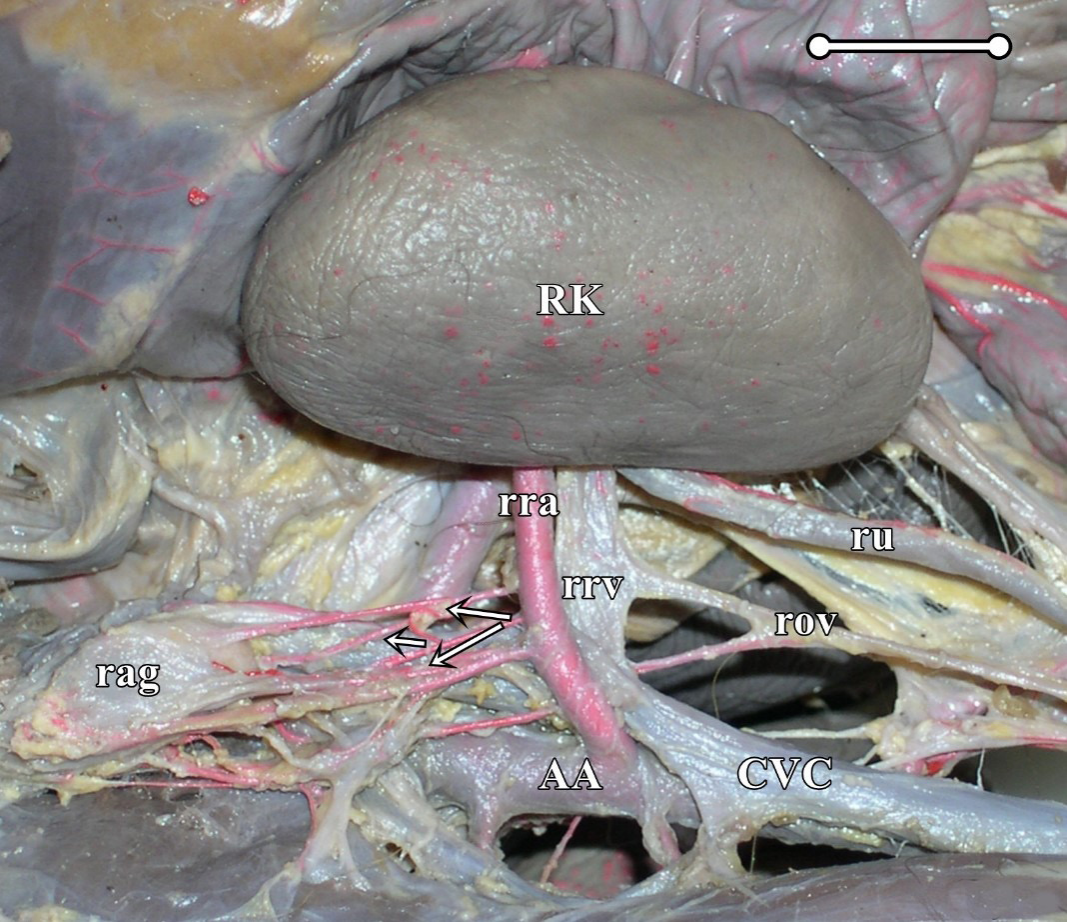
Digital photomacrography of the right lateral view of a kidney and renal vessels in a female specimen of *Alouatta guariba clamitans*. RK, right kidney; AA, abdominal aorta; CVC, caudal vena cava; rra, right renal artery; rag, right adrenal gland; rrv, right renal vein; rov, right ovarian vein; ru, right ureter; adrenal branches (arrows). Scale bar: 1.0 cm.

In all specimens, the renal arteries emerged from the abdominal aorta as a single vessel posterior to thauto-compe renal veins. After emerging from the abdominal aorta, they issued branches to the adrenal gland, ureter, diaphragm, and lumbar musculature before entering the hilum of the kidneys. The renal veins drained directly into the caudal vena cava as a single vessel in all specimens. The left renal vein received a gonadal branch (Figure 2).

For both sexes, measures of length, width, and thickness of the kidneys and renal vessels were obtained (Table 1), as well as their skeletopy (Table 2).

**Table 1:** Mean and standard deviation of renal and vascular measurements (cm) separated by sex in *Alouatta guariba clamitans*.

	***Alouatta guariba***	
	**Males (n=2)**	**Females (n=2)**
**Length of the right kidney**	4.692 ± 0.052	3.945 ± 0.167
**Length of the left kidney**	4.605 ± 0.005	4.065 ± 0.253
**Width of the right kidney**	2.115 ± 0.003	1.660 ± 0.151
**Width of the left kidney**	2.231 ± 0.031	1.801 ± 0.292
**Thickness of the right kidney**	2.833 ± 0.003	2.037 ± 0.236
**Thickness of the left kidney**	2.709 ± 0.059	2.297 ± 0.243
**Ellipsoid volume of the right kidney**	1.491 ± 0.000	0.712 ± 0.174
**Ellipsoid volume of the left kidney**	1.506 ± 0.006	0.911 ± 0.292
**Length of the right renal artery**	3.613 ± 0.063	1.874 ± 0.280
**Length of the left renal artery**	2.440 ± 0.035	2.172 ± 0.112
**Length of the right renal vein**	0.523 ± 0.013	1.331 ± 0.190
**Length of the left renal vein**	1.249 ± 0.049	3.185 ± 0.056

**Table 2:** Absolute frequency and simple percentage of right and left kidney skeletopy in *Alouatta guariba clamitans*.

**Skeletopy**	**Males (n=2)**				**Females (n=2)**			
	**Right**		**Left**		**Right**		**Left**	
**T13–L2**	2	100%	-	-	2	100%	-	-
**L2–L4**	**-**	-	2	100%	-	-	2	100%

In males, the left renal artery emerged uniquely directly from the aorta at the L3 level. The right renal artery emerged uniquely directly from the aorta at the L1 level. The left renal vein drained into the caudal vena cava at the L3 level. The right renal vein drained into the caudal vena cava at the L1 level.

In females, the left renal artery emerged directly from the aorta at level L2 in one specimen and L3 in another. The right renal artery emerged directly from the aorta at the L1–L2 level in one specimen and L2 in another. The left renal vein drained into the caudal vena cava at the L2 level in one specimen and L3 in another. The right renal vein drained into the caudal vena cava at the L1–L2 level in one specimen and L2 in another.

## Discussion

In *A. g. clamitans*, the kidneys have a symmetrical shape, which resembles a bean with a smooth surface without lobation, similar to that observed in common marmosets (*C. jacchus*) (Marini et al., 2018). du Plessis et al. (2017), in a computed tomography study with common marmosets, and Wagner and Kirberger (2005), in ultrasonographic examinations, observed that both kidneys were oval shaped.

In captive ring-tailed lemurs (*Lemur catta*), Makungu et al. (2016) observed that on ultrasonography, the kidneys appeared ovoid on both transverse and longitudinal images. However, on right lateral radiography, the left kidney was frequently bean shaped and rarely ovoid, whereas the right kidney was either ovoid or bean shaped.

In an ultrasonographic study with rhesus monkeys (*Macaca mulatta*) in the transverse scan, the kidneys were round in shape, whereas in the longitudinal scan, the outline was ovoid (James Junior et al., 1976).

Lima et al. (2016) observed that the kidneys of capuchin monkeys (*Sapajus apella*) presented different shapes, with the right kidney being similar to a bean and the left kidney having a tapered lower end, rounded upper end, and no lobation.

In cynomolgus monkeys (*M. fascicularis*), the shapes of the right and left kidneys were not identical. The left kidney had a smaller, more tapered cranial pole with a broad and round caudal pole. In contrast, the right kidney is uniformly oval, resembling a human kidney (Gaschen et al., 2000).

In an ultrasound assessment performed in squirrel monkeys (*Saimiri collinsi*) (Lins e Lins et al., 2017), both kidneys in all animals showed a regular elliptical shape, similar to that observed by Lins e Lins et al. (2012) in *Aotus azarai infulatus*.

In longitudinal and transverse sections, the kidneys of *A. g. clamitans*. consist of an outer renal cortex and an inner medulla with a pelvis and renal crest. The kidneys are simple and unipyramidal. These characteristics were also observed in *C. jacchus* (Marini et al., 2018), the cynomolgus monkeys (*Macaca fascicularis*) (Chamanza et al., 2019), capuchin monkeys (*Sapajus apella*) (Lima et al., 2016), and *Chlorocebus sabaeus* (Amory et al., 2013). However, the kidney of the spider monkey (*Ateles geoffroyi*) is unique because it is multipyramidal (Goodman et al., 1977).

Despite the small sample size, observing asymmetry of position in the kidneys was possible, with the right kidney being more cranial than the left. Knowledge of the location of kidneys is of paramount importance in radiological, ultrasonographic, surgical, and clinical procedures. This more cranial positioning of the right kidney was also observed in an ultrasound assessment performed in squirrel monkeys (*Saimiri collinsi*) (Lins e Lins et al., 2017), *Aotus azarai infulatus* (Lins e Lins et al., 2012), captive ring-tailed lemurs (*Lemur catta*) (Makungu et al., 2016), and *Chlorocebus sabaeus* (Amory et al., 2013).

According to a computed tomography study of common marmosets (*Callithrix jacchus)* by du Plessis et al. (2017), both kidneys were positioned between L1 and L3. The right kidney was positioned cranially to the left kidney in four of the eight animals, and the kidneys were at the same level in four of the eight animals. However, in a radiographic study by Wagner and Kirberger (2005) with common marmosets (*Callithrix jacchus*), the right kidney was typically positioned caudally to the left; however, no statistical difference was noted in kidney position between females and males. The difference observed in this study in the location of the kidneys may be related to the fact that specimens of *Callithrix* spp. are hybrids, presenting a different morphology from that of *C. jacchus.*

Moreover, in howler monkeys (*A. fusca*), Sartor et al. (2017) only commented that the right kidney is more cranial than the left, without providing details of the skeletopy.

In all specimens in this study, the renal arteries emerged from the abdominal aorta as a single vessel and emitted branches to the adrenal gland and ureter branches before entering the kidney. Furthermore, the renal veins drained directly into the caudal vena cava as a single vessel.

## Conclusion

The kidneys of *A. g. clamitans* resemble a “bean seed.” The medullary region is unipyramidal, and the ureter originates from the lower portion of the renal pelvis. The right kidney has a more cranial origin than the left. The renal arteries emerge from the abdominal aorta as a single vessel posterior to the renal veins. The renal veins drain directly into the caudal vena cava as a single vessel in all specimens.
